# Supplementary material for: Association of sarcopenia with important health conditions among community-dwelling Asian women
Source: PLoS One. 2023 Jan 30;18(1):e0281144. doi: 10.1371/journal.pone.0281144 (PMC9886252; doi:10.1371/journal.pone.0281144)
Supplement: S1 Table — (PDF) [file pone.0281144.s001.pdf]

S1 Table. Assay parameters for individual variables (n=1201).

	Concentration Range	Intra-assay coefficient of variance (%)	Inter-assay coefficient of variance (%)
Glucose (mmol/L)	2.1 - 24.4	0.6 - 1.6	0.8 - 2.0
25 (OH)D_2 (ng/mL)	1.0 - 80.0	3.4 – 6.7	5.4 – 9.1
Hs-CRP (mg/L)	1.0 - 69.4	0.5 - 1.4	1.0 - 1.6
IL-6 (pg/mL)	89.0 - 724.0	3.3 - 4.9	4.6 - 7.2
TNF- α (pg/mL)	91.0 – 526.0	6.3 - 6.6	3.3 - 4.5
