# Supplementary material for: Association of sarcopenia with important health conditions among community-dwelling Asian women
Source: PLoS One. 2023 Jan 30;18(1):e0281144. doi: 10.1371/journal.pone.0281144 (PMC9886252; doi:10.1371/journal.pone.0281144)
Supplement: Attachment — Submitted filename: Response to Reviewers.pdf [file pone.0281144.s002.pdf]

Manuscript ID [PONE-D-22-13016] "Association of sarcopenia with important health conditions among community-dwelling Asian women"

<u>RESPONSE TO EDITOR</u>

We would like to amend our Funding Statement to now read: "This study was funded by the Singapore National Medical Research Council Grant (Reference number: NMRC/CSA-SI/0010/2017) for ELY. There was no additional external funding received for this study."

<u>RESPONSE TO REVIEWER 1</u>

This is a very well written and well presented manuscript. I had only one comment for the authors to consider: Regarding the sentence: "Chinese women made up the majority 159 at 81.1% (Table 1), while Malay and Indian women constituted 5.5% and 9.9% respectively," the percentages do not add up to 100%. It may be useful to state that in this sentence to help the reader understand that this is due to missing data.

<u>Response:</u> We thank reviewer 1 for his/her kind comments. We have added the following sentence in lines 189-190 of the tracked changes document: *"The remaining 3.5% constituted of women from other ethnicities."*

<u>RESPONSE TO REVIEWER 2</u>

I believe that this type of research has been conducted so frequently that it does not provide particularly useful or novel information.

<u>Response:</u> The novelty of our research is that we examined several important health conditions specific to midlife women to provide a holistic view of the relationship between these health conditions and sarcopenia. As mentioned in lines 33-39 of the tracked changes document: *"In addition, most studies globally (8, 9) and in Singapore (14, 15) focused mainly on the relationship between sarcopenia and single health conditions. In this respect, the Integrated Women's Health Program (IWHP) has identified important health conditions that manifest in midlife women, including high systolic blood pressure (20), insulin resistance (21), osteoporosis (22), depression and anxiety (23), and urinary incontinence (24)."*

Most sarcopenia studies were conducted in Caucasian populations and did not focus on midlife women. We found it particularly valuable to conduct such studies in midlife women as mentioned in lines 29-32: *"and there is some evidence that muscle mass reduction accelerates during the time around menopause (18). Decrease in estrogen levels post-menopause have been associated with a decrease in muscle mass and function (18, 19)."*

Therefore, it would be more accurate to refer to this as a conceptual study that evaluates the relationship between specific diseases and potential new risk factors in a well-planned prospective cohort, rather than a study of the relationship between various health conditions.

<u>Response:</u> Rather than being conceptual, this paper presents empirical data to examine the relationship of sarcopenia, as defined by AWGS and FNIH, with important health conditions affecting midlife women using objectively measured parameters and validated questionnaires.

Please explain the cohort definition in more detail.

<u>Response:</u> To further define the cohort, we have included the following in lines 54-70: *"The cohort has been fully described previously (25). Briefly, healthy women aged 45-69 years receiving routine gynecological follow-up at well-women clinics at the National University Hospital were recruited from September 2014 to October 2016 through fliers, posters, and word-of-mouth. Exclusion criteria included pregnancy or being severely ill. The number of*

women screened was at 2715, and 2191 met the eligibility criteria. Thereafter, 746 subjects, 134 subjects and 110 subjects declined participation, failed to attend the appointment and were non-contactable respectively, leaving a final sample of 1201 women (25). The protocol was approved by the Domain Specific Review Board of the National Healthcare Group, Singapore (Reference number: 2014/00356) and all participants provided written informed consent. In short, a whole-body composition scan was performed, and a fasting blood sample was collected for participants on arrival. This was followed by light refreshments, a series of questionnaires, biophysical measurements, and physical function tests, with the visit approximating 90 minutes."

Please draw and explain the flow chart for the subjects included in the final study in the study target group.
Response: The flowchart for the current cohort has been previously published in reference (25). To give a clearer overview of subjects enrolled in this study, we have additionally included the following sentence in lines 59-63: *"The number of women screened was at 2715, and 2191 met the eligibility criteria. Thereafter, 746 subjects, 134 subjects and 110 subjects declined participation, failed to attend the appointment and were non-contactable respectively, leaving a final sample of 1201 women."*

The average age of the study population is too young to study sarcopenia.
Response: In lines 25-26, we have acknowledged that *"studies on sarcopenia were mostly conducted in elderly subjects."* Nevertheless, this study focuses on midlife subjects for the following reasons mentioned in lines 26-33: *"Nevertheless there is increasing realization that the development of sarcopenia may start earlier in life (16), emphasizing the need to study sarcopenia across the life course. Women have lower absolute muscle mass than men (17), and there is some evidence that muscle mass reduction accelerates during the time around menopause (18). Decrease in estrogen levels post-menopause have been associated with a decrease in muscle mass and function (18, 19). Hence, studies on sarcopenia should also focus on younger women as much as in geriatric men."*

RESPONSE TO REVIEWER 3

This is a good manuscript, well written, very clear, with a high sample, with a clear message and appropriate statistics. Unfortunately this is a cross sectional study and the association between sarcopenia and other pathologies remains difficult to interpret.
Response: We thank the reviewer for his/her kind comments about the manuscript. We agree that this cross-sectional study would not be able to dissect the cause-and-effect relationship between sarcopenia and other health conditions, and this has been mentioned from lines 336-338 of the tracked changes document: *"A limitation of our study is the cross-sectional design, thereby precluding any conclusions as to causality or temporality. We are unable to ascertain if sarcopenia leads to T2DM and osteoporosis, or vice versa."*
To address this limitation, we have almost completed our 7-year follow-up of the cohort, and the conclusions of this follow-up study would be published soon, as stated in lines 338-341, *"However, longitudinal follow-up visits in our cohort are underway and will clarify the relationship between sarcopenia and incident health conditions as women age."*

Authors report data of MA on falls and mortality. I know that there are other high-quality SR/MA reporting data over other outcomes of sarcopenia. Please cite some of them to offer a more accurate bibliography.

Response: We have included *"functional decline, reduced quality of life"* and *"cardiovascular diseases*" as other outcomes of sarcopenia, citing three additional papers from high-quality journals for a more accurate bibliography. Lines 2-5 state that: *"Sarcopenia, the loss of skeletal muscle mass with advancing years, is associated with increased risk of falls (1), functional decline, reduced quality of life (2), higher hospitalization rates and related costs (3), cardiovascular diseases (4), disability (5), and even mortality (5, 6)."*

Why did the authors chosen the FNIH definition to be compared with AWGS? Why only reporting prevalence for those two particular definitions?
Response: We agree that there are many definitions of sarcopenia. We chose the AWGS as it is most appropriate for Asian women. Furthermore, the AWGS definition is similar to, being a derivative of the European Working Group on Sarcopenia in Older People (EWGSOP). We included the FNIH definition as it is commonly used by sarcopenia researchers globally.

The secondary objectives of this paper (i.e. measuring associations of sarcopenia with hypertension, T2DM, osteoporosis, depression and anxiety) have not been introduced. Please provide a rationale for those analyses.
Response: To make the secondary objectives of the paper clearer, we have mentioned the following in lines 35-45: *"In this respect, the Integrated Women's Health Program (IWHP) has identified important health conditions that manifest in midlife women, including high systolic blood pressure (20), insulin resistance (21), osteoporosis (22), depression and anxiety (23), and urinary incontinence (24). Since hypertension, T2DM, osteoporosis, depression and anxiety, and urinary incontinence are the most common and critical health conditions, we studied their association with sarcopenia as these conditions are likely to progress and result in a higher healthcare burden on midlife women as they age. Identification of the link between sarcopenia and these common health conditions would allow for specific preventive healthcare initiatives."*
Method: Please follow STROBE statement and report the use of this checklist in the methods section.
Response: The design of our study follows all the items in the STROBE statement, and this is now indicated in line 74-75: *"This study was performed in concordance with all the items required in the STROBE statement (26)."*

Did authors calibrated the DXA and Jamar?
Response: Yes, we calibrated the DXA and Jamar on a regular basis. We have added the following information in lines 110-114: *"Operators of the DXA machine were trained to follow standard protocols according to the manufacturer's instructions. The DXA machine was calibrated daily and sent for maintenance every 6 months. Handgrip strength was assessed using a dynamometer (Jamar, Bolingbrook, IL), which was calibrated yearly."*

How did authors measured the normality of variables?
Response: We have added the information in lines 160-161: *"The normality of the variables was examined visually using histograms and Q-Q plots, as well as using the Kolmogorov-Smirnov and Shapiro-Wilk tests."*

This is surprising to have such a high prevalence of sarcopenia in health community dwelling participants so young (45-69 years). Do you have an hypothesis to explain this high prevalence?

Response:

We have now included the following reasons to possibly explain the high prevalence of sarcopenia in our young participants in lines 253-262: *"The prevalence of AWGS sarcopenia in our cohort (18.0%) and in another recent Singapore study (14.2% in women, aged 21-90 years) (12) was higher than that of other Asian populations in Taiwan (3.7% in women, mean age: 63.3 ± 10.0 years) (39) and Korea (13.8% in women, aged 70 to 84 years) (40). A probable reason for the relatively higher sarcopenia prevalence in Singapore might be due to the warmer tropical climate, serving as a barrier to physical activity (41). The well-developed transport infrastructure in Singapore might have reduced opportunities for active commuting, which is known to improve exercise capacity and cardiovascular outcomes (42)."*

Could you also tell a bit more about the representativity of the sample?

Response: Our cohort closely reflects the demographics of women in Singapore and we have added that in lines 356-362: *"The findings from our study are generalizable to the general population in Singapore as our participants' demographics were comparable to the rest of the population in terms of ethnicity (81% Chinese in our cohort vs 78% in the general population) and highest education level attained (20.2% university level qualification in our cohort vs 16.4% in the population) (61). Our findings will also be generalizable to other midlife women in urbanized Asian cities."*

I would be interesting also to know how many among the 93 individuals diagnosed as sarcopenic using the FNIH criteria also are diagnosed sarcopenic with the AWGS criteria.

Response: In the Venn diagram (Figure 1), we have indicated that out of 93 individuals diagnosed as sarcopenic using the FNIH criteria, n=50 were also diagnosed as sarcopenic with the AWGS criteria as seen from the overlapped region between the two circles.

Please report 95%CI for the Kappa agreement between both diagnoses.

Response: We have reported the 95% CI for the Kappa agreement in line 195: *"Cohen's κ of 0.24 (95%CI: 0.17, 0.31) indicated poor agreement between the two criteria."*

In Table 2, please remove the "adjusted" OR since some of them were crude OR.

Response: We have removed the word "adjusted" in table 2.
